# The association between workplace bullying and depressive symptoms: the role of the perpetrator

**DOI:** 10.1186/s12889-016-3657-x

**Published:** 2016-09-17

**Authors:** Eszter Török, Åse Marie Hansen, Matias Brødsgaard Grynderup, Anne Helene Garde, Annie Høgh, Kirsten Nabe-Nielsen

**Affiliations:** 1 Section of Social Medicine, Department of Public Health, University of Copenhagen, Øster Farimagsgade 5, 1014 Copenhagen, Denmark; 2 National Research Centre for the Working Environment, Lersø Parkallé 105, 2100 Copenhagen, Denmark; 3 Department of Psychology, University of Copenhagen, Øster Farimagsgade 2A, 1353 Copenhagen, Denmark

**Keywords:** Workplace bullying, Perpetrator, Depressive symptoms, Major Depression Inventory (MDI)

## Abstract

**Background:**

The aim of the present study was to investigate whether the depressive symptoms of the bullied respondents differed according to who the perpetrator was.

**Methods:**

We used cross-sectional questionnaire data from two representative cohorts: the Danish Working Environment Cohort Study (DWECS 2010) and the Work and Health Study (WH 2012). After excluding respondents not having a leader, or being self-employed, assisting spouses, and those reporting multiple perpetrators in WH 2012, the statistical analysis included 2478 bullied individuals. We compared respondents reporting being bullied by their (1) leader, (2) subordinates, (3) clients / customers / patients / students, or (4) colleagues, respectively. The occurrence of depressive symptoms was measured by the Major Depression Inventory (MDI).

**Results:**

The most frequent perpetrator of bullying was clients (41.5 %) in DWECS 2010 and colleagues (60.3 %) in WH 2012. In DWECS 2010, the MDI score of those being bullied by clients were significantly lower than the MDI scores of the other groups. In WH 2012, respondents who reported bullying from leaders had a significantly higher mean MDI score than participants being bullied by colleagues. Also in WH 2012, our results indicated that those who were bullied by leaders had a higher MDI score than those bullied by clients, although this difference was not statistically significant at conventional levels.

**Conclusion:**

Our findings indicated a similar pattern in the two cohorts, with a tendency of more severe depressive symptoms among employees who are exposed to bullying by their leaders, and the least severe symptoms among those who are bullied by clients.

## Background

Workplace bullying is defined as a situation where an individual, repeatedly over a prolonged period of time, is exposed to negative acts from one or several others, and where the target finds it difficult to defend him- or herself against these actions [1–3]. The prevalence of bullying shows large variations, depending on the operational criteria used to measure bullying [4–6]. When the definition of bullying is provided to the respondents, the prevalence of workplace bullying mostly varies between 2 % and 17 % [3], whereas without the definition, or using the behavioural experience method, i.e. the perception of being exposed to different types of negative acts, the prevalence is somewhat higher, varying between 15 % and 20 % [5, 7].

According to the literature, workplace bullying is mostly carried out by colleagues and leaders [6] with variations in the prevalence across countries. Whereas most studies in the US, UK and Europe consistently found that 50-70 % of all bullying cases are involving leaders [8–10], in Scandinavia (especially in Denmark, Sweden and Finland) colleagues are the most commonly reported perpetrators of bullying [1, 6, 11, 12]. This variation might be explained by cross-cultural differences concerning social relations, organisational culture, and work-related values and attitudes [1, 13, 14].

As regards health consequences, the literature consistently supports a negative effect of bullying on psychological health. Longitudinal studies with 1-2-year follow-up have shown that bullying is associated with a range of negative psychological health outcomes, such as psychological distress, sleep difficulties, depression and symptoms of anxiety [15–19]. In addition, studies also revealed long-term effects, as those experiencing social exclusion at their workplace had an increased risk of having symptoms of mental disorders even after 3-6-year follow-up [15, 20, 21]. Likewise, a study investigating workplace bullying and depressive symptoms in junior physicians found an increased risk of depressive symptoms in a three year follow-up [22]. Two Danish studies, furthermore, found an increased risk of depression among frequently bullied respondents compared to those occasionally bullied [16, 23]. Similarly, a recent meta-analysis [24] including only longitudinal studies estimated that the odds of mental health symptoms among bullied compared to non-bullied employees is 1.68 (95 % CI: 1.35-2.09).

In a few previous studies it has been proposed that the effects of the exposure to offensive behaviours such as bullying and other types of harassment may depend on the target’s interpretation of the acts [25, 26]. For example, it has been suggested that working as a nurse knowing that patients with psychiatric disorders may behave inappropriately could be interpreted in a different light than exposure to offensive behaviours from colleagues [27]. In line with this argument, previous studies have shown that in the eldercare services, being bullied by colleagues and leaders is more strongly associated with the risk of turnover [27, 28] compared to the risk associated with exposure to violence, threats, and unwanted sexual attention, in which cases the perpetrators are often care recipients with impaired mental capacities [29]. Similarly, other studies showed that the more formal power the perpetrator has over the target, the more likely the target experiences the harassing behaviour negatively [30, 31].

The Cognitive Activation Theory of Stress (CATS) [32] could contribute to the theoretical explanation of why the targets of workplace bullying may respond differently depending on the perpetrator. The core element of CATS is the expectancy of the outcome of stimuli, which can be positive (coping), none (helplessness), or negative (hopelessness) [32]. On the one hand, in case of exposure to bullying by a leader, the targets may perceive that regardless of what they do, they cannot predict and control the outcome (helplessness) or that whatever they do the situation will worsen (hopelessness). On the other hand, in case of being bullied by someone lower in the formal organisational hierarchy (e.g. subordinates or clients), the targets may perceive more control over the situation by having positive outcome expectancies (coping) in terms of support from the leader to stop the bullying or to be relocated within the workplace. Consequently, we suggest that targets who are bullied by leaders will not expect to be able to handle the situation with a positive result, which may damage their psychological health to a higher extent as compared to those who can cope with bullying.

Against this background, we suggest that the level of depressive symptoms resulting from workplace bullying may depend on who the perpetrator is. To our knowledge, no previous studies have investigated the relationship between the perpetrator and health outcomes of the targets in terms of depressive symptoms in the general working population. The aim of the present study, therefore, was to investigate whether the depressive symptoms of the bullied respondents differed according to who the perpetrator was. More specifically, we compared the level of depressive symptoms among those reporting having been bullied at their workplace by leaders, colleagues, subordinates or clients (here: clients, patients, customers, or students), respectively. We hypothesized that (1) those bullied by leaders reported more severe depressive symptoms compared to the other groups and (2) those bullied by clients reported the least severe depressive symptoms. We also expected those being bullied by colleagues and subordinates to be distributed between the other two groups, yet we did not have any predefined hypothesis about the level of their depressive symptoms relative to each other.

## Methods

### Study design and study population

Concern has been raised about differences between employees labelling themselves as being bullied as compared with those who do not label themselves as being bullied (e.g. in terms of personality and mental health) [1, 33–35]. Thus, the main analysis of the present study only included participants who labelled themselves as targets of bullying, whereas in an additional analysis we compared the depressive symptoms of the bullied and non-bullied respondents. To obtain a sufficiently large study population, we included questionnaire data from two cross-sectional surveys of the general working population in Denmark: we used self-reported questionnaire data from the Danish Working Environment Cohort Study in 2010 (DWECS 2010) and the Work and Health Study in 2012 (WH 2012). Although the two questionnaires were not identical, they were both appropriate for the purpose of the current study.

#### DWECS 2010

The questionnaire included 62 questions about the working environment and health. The DWECS 2010 is based on 30,000 randomly selected individuals aged 18 to 59 from the *general population* residing in Denmark. The sample was selected in early September 2010. In October 2010, the randomly selected people were sent a questionnaire by mail with an invitation to participate in the survey. They were offered the opportunity to choose between a paper and an online version of the questionnaire. Those not responding to the first request were contacted again with both a paper and an online version of the questionnaire. In case of no response after the second reminder, people were contacted via telephone, and were encouraged to participate in the survey as well as offered to get a new questionnaire sent. Overall, 14,453 people answered the questionnaire, yielding a response rate of 48 %, yet only 10,605 of the participants were currently employed, and therefore eligible for inclusion in the present study. Of these, 47 % were males and 53 % were females.

#### WH 2012

The questionnaire contained 55 main questions on occupational safety and health as well as a few questions regarding drinking, smoking and physical exercise. The study is based on a sample of the *working population* - a total of 35,000 people aged 18 to 64 years with residence in Denmark. A randomly sampled 35,000 people received a letter in April 2012 with an invitation to participate in the survey. Those not responding to the first request were contacted again with both an online and a paper version of the questionnaire. In case they still did not respond, they were contacted via telephone and were encouraged to participate in the survey as well as offered to get a new questionnaire sent. Overall, 16,412 employed people chose to answer the questionnaire, yielding a response rate of 47 %. Of these, 46 % were males and 54 % were females.

In DWECS 2010 and WH 2012, respectively, 9.7 % (*n* = 1028) and 11.9 % (*n* = 1961) of the respondents had been exposed to workplace bullying. We found that 96.4 % in the DWECS 2010 and 92.4 % in WH 2012 had been employed at their current workplace for more than 12 months. These results imply that the majority of the respondents were working at the workplace where the bullying occurred while filling out the questionnaire.

### Exclusion criteria

For the purpose of the present study, we excluded participants who did not label themselves as being bullied (*n* = 23,474) in both cohorts. Among the remaining participants (*n* = 3543), we aimed at including respondents who were potentially at risk of being bullied by any of the perpetrators presented in the surveys. Therefore, participants reporting not having a leader (*n* = 102) were excluded from the analysis, assuming that they were not being at risk of being bullied by a leader. Similarly, we also excluded participants who were self-employed (*n* = 66) or working as assisting spouses (*n* = 4), assuming that the former group did not have a leader, and the latter group had neither a leader nor colleagues in the formal sense. The data sets, however, did not include any information about respondents not having clients and subordinates. In addition, from the main analyses we excluded participants reporting multiple perpetrators in WH 2012 (*n* = 313), of whom 254 reported bullying by leaders, 286 by colleagues, 78 by clients and 44 by subordinates. Overall, we included data from 2478 bullied individuals (DWECS 2010: *n* = 958; WH 2012: *n* = 1520).

### Study variables

#### Workplace bullying

##### DWECS 2010

The prevalence of workplace bullying was assessed by the following question: “Have you over several months been exposed to unpleasant or degrading treatment that was hard to defend yourself against?” Respondents were asked to report whether they had been exposed to workplace bullying within the last 12 months, by asking them to choose one of the following response categories: (a) No; (b) Yes, from colleagues; (c) Yes, from a leader; (d) Yes, from subordinates; (e) Yes, from clients / customers / patients / students.

##### WH 2012

The prevalence of workplace bullying was assessed using the following definition: “Bullying takes place when a person repeatedly, over a long period of time, is exposed to one or more persons’ offensive acts that the person perceives as hurtful or degrading.” Respondents were asked to indicate whether they had been exposed to bullying at work within the last 12 months, by asking them to choose from the following options: (a) Yes, daily; (b) Yes, weekly; (c) Yes, monthly; (d) Yes, now and then; or (e) Never. In the subsequent question, those being exposed to workplace bullying were asked to indicate the perpetrator of bullying: (a) colleagues; (b) leader; (c) subordinates; and (d) clients / customers / patients / students. (For simplicity, the latter category will be referred to as “clients” in the remaining part of the paper.) For this question, the respondents could choose more than one category, whereas this option was not provided in DWECS 2010. Therefore, we could not investigate the health outcomes of those being exposed to bullying by multiple perpetrators in the total sample, and thus in WH 2012 only those respondents were included who were bullied by only one perpetrator. As an additional analysis, however, we compared the health outcomes of respondents being bullied by multiple perpetrators with those reporting one perpetrator in the WH 2012 sample.

#### Depressive symptoms

The occurrence of depressive symptoms among the respondents was measured by the Major Depression Inventory (MDI) [36]. According to the Schedule for Clinical Assessment in Neuropsychiatry (SCAN), the sensitivity of the MDI for major depression varies between 0.86 and 0.92, and its specificity varies between 0.82 and 0.86 [36]. The MDI is a self-rated questionnaire consisting of 10 items, of which two have a sub-item. Thus, in total the MDI contains 12 items measuring the presence of core and accompanying symptoms of depression during the past 2 weeks on a scale ranging from 1 = All the time to 5 = No, never. The overall scores of the MDI range from 0 to 50 with an optimal cut-off score of 26 indicating major depression [36]. In the preliminary analyses we found that 9 % of the bullied respondents reached the cut-off score for major depression in both cohorts. Thus, using major depression as the outcome while also splitting the exposure into sub-categories would have left us with insufficient statistical power. Therefore, in the present study including the general working population, we did not use this cut-off score; instead we treated the MDI as a continuous variable measuring depressive symptoms.

#### Sociodemographic factors

Information about gender, age and occupational sector was obtained from the Danish Civil Registration System (Table 1).

**Table 1:** Overview of the bullied respondents in DWECS 2010 and in WH 2012: distribution of occupational sectors, age, gender, and the mean scores of the Major Depression Inventory (MDI)

	DWECS 2010	WH 2012
Occupational sectors		
Industry	85 (8.9 %)	205 (15.7 %)
Construction	20 (2.1 %)	51 (3.9 %)
Graphical	10 (1.0 %)	11 (0.8 %)
Transportation	79 (8.2 %)	156 (12.0 %)
Trade	28 (2.9 %)	52 (4.0 %)
Services	52 (5.4 %)	140 (10.7 %)
Farm	11 (1.1 %)	29 (2.2 %)
Social and Health	229 (23.9 %)	331 (25.4 %)
Education and Research	74 (7.7 %)	151 (11.6 %)
Finance	64 (6.7 %)	92 (7.0 %)
Private office and administration	43 (4.5 %)	87 (6.7 %)
Unknown^a^	263 (27.5 %)	0 (0 %)
Age (Mean, SD)	43.4 (11.3)	46.0 (11.1)
MDI (Mean, SD)	11.0 (9.4)	12.8 (9.1)
Gender		
Male	391 (40.8 %)	628 (41.3 %)
Female	567 (59.2 %)	892 (58.7 %)

^a^Due to the coding procedure in DWECS 2010, we were not able to get information about these participants concerning their occupation

### Statistical analysis

All statistical analyses were conducted using IBM SPSS Statistics 21. Frequency analysis was used to investigate the prevalence of the reported perpetrators. In a one-way ANOVA we tested the difference between the MDI scores among those bullied by leaders, colleagues, subordinates and clients, respectively. As equal variances in the MDI scores between the groups were not confirmed, Games-Howell pairwise multiple comparisons were conducted. Finally, an ANCOVA analysis was performed in order to test whether the variance of the MDI scores could be explained by the perpetrator when adjusting for age, gender, and occupational sector. A p-value < 0.05 was considered statistically significant.

In order to increase the statistical power of the study, we aimed at merging data from DWECS 2010 and WH 2012. In an initial analysis of whether the MDI score of the targets depended on who the perpetrator was, we found a statistically significant difference between the surveys (survey*perpetrator interaction *p* = .022) when taking the distribution of gender, age, and occupational sector into account. Since our initial analyses indicated a difference in the effect of the perpetrators on the MDI scores between the two surveys, we subsequently decided to analyse and present the results separately, instead of merging the data.

## Results

### Descriptive data

The majority of the respondents belonged to the social and health care sector both in DWECS 2010 (*n* = 229, 23.9 %) and in WH 2012 (*n* = 331, 25.4 %), representing the biggest group of the study, whereas the least amount of respondents belonged to the graphical sector (*n* = 10, 1 % in DWECS 2010, *n* = 11, 0.8 % in WH 2012), representing the smallest group. The distribution of occupational sectors, age, gender and depressive symptoms (MDI) of the bullied respondents are presented in Table 1.

### DWECS 2010

The results of DWECS 2010 showed that the most commonly reported perpetrators of workplace bullying were clients (41.5 %), whereas the least common perpetrators were subordinates (4.2 %). The prevalence of being bullied by leaders and colleagues were 23.8 % and 30.5 %, respectively. The mean MDI score was the lowest (mean: 8.9; SD: 8.2) among those being bullied by clients, and it was the highest (mean: 13.1; SD: 9.6) among those being bullied by subordinates (Table 2).

**Table 2:** Overview of DWECS 2010 and WH 2012: characteristics of the bullied respondents, and the prevalence of being bullied by one of the four perpetrator groups

Characteristics of the targets			Reported perpetrator															
			Leader				Colleague				Subordinate				Client			
			N	%	Mean	SD	N	%	Mean	SD	N	%	Mean	SD	N	%	Mean	SD
DWECS 2010	Gender	Male	81	20.7		.	104	26.6	.	.	23	5.9	.	.	183	46.8	.	.
		Female	147	25.9	.	.	188	33.2	.	.	17	3.0	.	.	215	37.9	.	.
	Age		-	.	44.7	11.1	-	.	43.3	11.1	-	.	45.8	10.4	-	.	42.6	11.6
	MDI		-	.	12.7	10.0	-	.	12.3	9.9	-	.	13.1	9.6	-	.	8.9	8.2
WH 2012	Gender	Male	156	24.8		.	385	61.3	.	.	31	4.9	.	.	56	8.9	.	.
		Female	243	27.2	.	.	531	59.5	.	.	37	4.1	.	.	81	9.1	.	.
	Age		-	.	46	10.6	-	.	45.7	11.3	-	.	46.8	10.4	-	.	47.7	11.4
	MDI		-	.	14	9.4	-	.	12.4	9.1	-	.	11.7	7.8	-	.	11.9	7.9

Table 3 reports the adjusted results concerning the mean MDI scores of the bullied respondents according to the perpetrators, and shows that the MDI score of those who were bullied by clients differs significantly from the other three groups. Compared to those being bullied by their clients, individuals who reported bullying from leaders (mean difference: 4.29; 95 % CI: 2.27-6.31), subordinates (mean difference: 4.41; 95 % CI: 0.33-8.49), and colleagues (mean difference: 3.55; 95 % CI: 1.64-5.46) had a significantly higher mean MDI score. No other statistically significant difference was found between the groups in this sample.

**Table 3:** Major Depression Inventory (MDI) scores of those being exposed to workplace bullying by leaders, colleagues, subordinates or clients: pairwise multiple comparisons of the MDI scores of the targets according to the reported perpetrators

Adjusted model	Reported perpetrator	N	Mean (MDI)	SD	Pairwise comparisons	Mean difference	95 % CI
DWECS 2010^a^	Leader	228	13.1	0.7	Subordinates	-0.12	[-4.34;4.09]
					Clients	4.29	[2.27;6.31]
					Colleagues	0.74	[-1.4;2.89]
	Colleagues	292	12.3	0.7	Subordinates	-0.87	[-5.02;3.28]
					Clients	3.55	[1.64;5.46]
	Subordinates	40	13.2	1.5	Clients	4.41	[0.33;8.49]
WH 2012^b^	Leader	399	14.1	0.6	Subordinates	1.90	[-1.39;5.18]
					Clients	2.39	[-0.25;5.03]
					Colleagues	1.67	[0.14;3.20]
	Colleagues	916	12.4	0.4	Subordinates	0.23	[-2.92;3.37]
					Clients	0.72	[-1.75;3.20]
	Subordinates	68	12.2	1.2	Clients	0.50	[-3.32;4.31]

^a^Model fit information after adjusting for age, gender and occupational sector: F(15, 1278) = 2.6, *p* = .001, ηp^2^ = .03

^b^Model fit information after adjusting for age, gender and occupational sector: F(16, 954) = 4.3, *p* = .000, ηp^2^ = .07

### WH 2012

The results of WH 2012 showed that the most commonly reported perpetrators of workplace bullying were colleagues (60.3 %), whereas the least common perpetrators were subordinates (4.5 %). The prevalence of being bullied by leaders and clients were 26.3 % and 9.0 %, respectively. The mean MDI scores were the lowest (mean: 11.7; SD: 7.8) among those being bullied by their subordinates, and it was the highest (mean: 14.0; SD: 9.4) among those being bullied by their leaders (Table 2).

Respondents being bullied by leaders had a significantly higher mean MDI score than participants being bullied by colleagues (mean difference: 1.67; 95 % CI: 0.14-3.20), yet no other significant difference regarding the MDI scores was found among the four perpetrator groups. Nevertheless, we also found a considerably higher MDI score among those being bullied by a leader (mean difference: 2.39; 95 % CI: -0.25-5.03) compared with those being bullied by clients, although the difference was not statistically significant at conventional levels (Table 3).

In addition, we compared the mean MDI scores of the non-bullied and bullied groups. In DWECS 2010 the results showed that non-bullied respondents had significantly lower mean MDI scores compared to those who were bullied by leaders (mean difference: -6.38; 95 % CI: -7.62- -5.15) colleagues (mean difference: -5.66; 95 % CI: -6.77- -4.55), subordinates (mean difference: -6.75; 95 % CI: -9.68- -3.82), and clients (mean difference: -2.21; 95 % CI: -3.16- -1.25). We found the same results in WH 2012, where non-bullied respondents reported lower MDI scores than those bullied by leaders (mean difference: -6.56; 95 % CI: -7.65- -5.48), colleagues (mean difference: -4.80; 95 % CI: -5.53- -4.07), subordinates (mean difference: -4.50; 95 % CI: -7.06- -1.93, and clients (mean difference: -4.22; 95 % CI: -6.16- -2.28).

As an additional analysis, we compared the mean MDI scores of respondents being bullied by one perpetrator (one perpetrator: *n* = 1520) with those who were excluded from the main analyses due to reporting multiple perpetrators (two perpetrators: *n* = 280; three or more perpetrators: *n* = 33). While adjusting for age, gender and occupational sector, we found that those reporting two perpetrators had a significantly higher mean MDI score than those reporting only one perpetrator (mean difference: 2.52; 95 % CI: 0.96-4.08). The difference between those reporting one versus three or more perpetrators was of a similar size, although it was not statistically significant due to the small numbers (mean difference: 2.46; 95 % CI:-1.78-6.69). There was no difference between those reporting two and those who reported three or more perpetrators (mean difference: 0.06; 95 % CI: -4.37-4.50).

### Comparison of DWECS 2010 and WH 2012

**Fig. 1:**
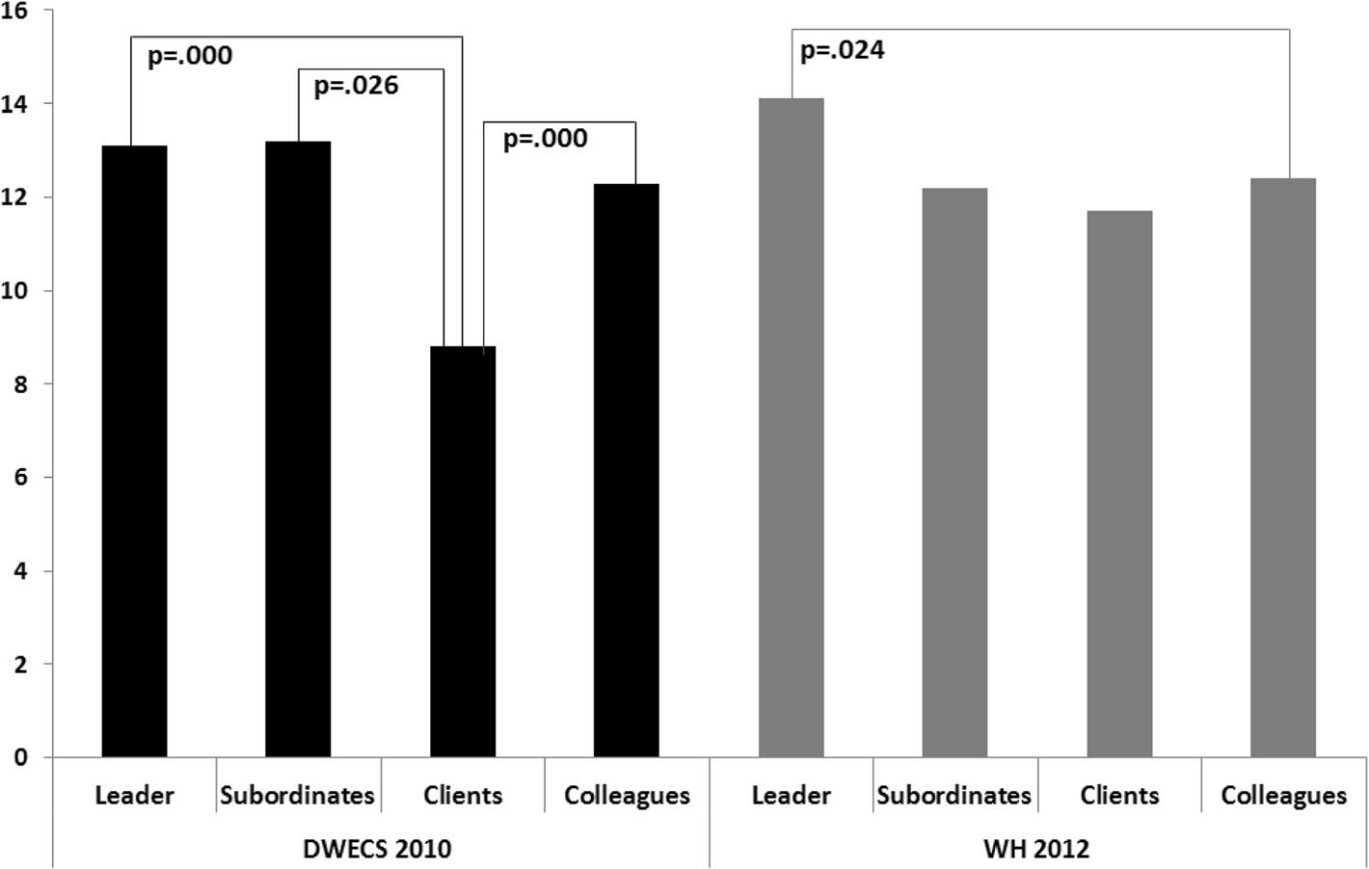
The reported perpetrators and the Major Depression Inventory (MDI) scores of the bullied respondents after controlling for age, gender and occupational sector in DWECS 2010 and in WH 2012

In DWECS 2010, the effect sizes of the significant relationships ranged from 3.55 to 4.41, whereas the effect size of the only significant association in WH 2012 was 1.67. These effect sizes can be quantified as up to 47 % of the standard deviation of the mean MDI score, although relative to the whole scale range (0-50) these differences are rather small. In order to make the pattern of DWECS 2010 and WH 2012 visually comparable, Fig. 1 illustrates the adjusted MDI scores of the bullied respondents depending on the self-reported perpetrators.

## Discussion

### Primary findings

In DWECS 2010 we found that those being bullied by their clients had significantly lower scores on the MDI scale compared to those bullied by leaders and colleagues. Likewise, in WH 2012 we found an indication that those bullied by clients had less severe depressive symptoms compared to those bullied by leaders, yet due to the few respondents in the client category this association was not statistically significant at the conventional level. Furthermore, in WH 2012 those bullied by their colleagues had significantly less severe depressive symptoms than those who were bullied by their leaders.

Taken together, the overall pattern of the MDI scores of those being bullied by leaders, clients and colleagues is alike in the two surveys, whereas the influence of being exposed to bullying by subordinates on the MDI scores did not show a clear pattern. As one cohort showed significant differences between the MDI scores of those bullied by clients and leaders, and the other cohort only showed a non-significant tendency, it is hard to draw a universal conclusion.

One reason for the observed differences in occurrence of participants being bullied by clients could be the different distribution of occupational sectors. However, the two surveys were based on random samples of the general working population and very similar in composition with regards to the included occupational sectors. In both cohorts a similar (and the biggest) proportion of the respondents belonged to the “Social and Health” sector, which is typically including the largest amount of clients. Thus, the difference in occurrence of participants being bullied by clients is not likely to be due to the occupational composition of the two surveys.

Another reason for the different association between the various perpetrators and the MDI scores of the bullied respondents could be the slight difference in the definition of bullying between the two surveys. Although the dissimilarity may have influenced our results, this explanation is not highly likely as the overall prevalence of bullying was similar between the two studies.

In general, we made a considerable effort (e.g. investigating the distribution of different occupational sectors, sampling procedures, or the formulation of the definition of bullying) in order to illuminate the explanation for the remarkable differences in the two cohorts, yet none of them resulted in a convincing explanation.

### Comparison with previous studies

We hypothesized that (1) those bullied by leaders reported more severe depressive symptoms compared to the other groups and (2) those bullied by clients reported the least severe depressive symptoms. Our hypotheses were only party confirmed by the results.

The results of DWECS 2010 support the findings of Clausen et al. [27], who proposed that bullying might be experienced less severely in case an employee is bullied by clients with impaired mental capacities e.g. in eldercare. However, in our study the response category “clients” also included customers, patients and students, and so could be interpreted more broadly, thus we cannot state that those who reported bullying from clients only provided service for patients with mental illnesses. Nevertheless, the result supports the notion that the less powerful the perpetrator is, the less severe the target’s depressive symptoms are [30]. Furthermore, in case of being bullied by clients, the targets may have the possibility to seek social support from their leader or colleagues in order to cope with bullying [37, 38].

Interestingly, the depressive symptoms of those who were bullied by their subordinates - as a group of less powerful perpetrators in terms of occupational hierarchy - were not any less severe than those being exposed to bullying by others. This finding goes somewhat against the role of the previously highlighted formal power in terms of health outcomes of bullying. Nevertheless, it may be explained by the Scandinavian organisational culture [1], in which the differences in power between individuals in various formal and informal positions are relatively small, and therefore being bullied e.g. by a leader or colleagues may have similar outcomes than being bullied by a subordinate. It is important to note, however, that due to the low prevalence of bullying by subordinates, we had low statistical power to test its difference from the other perpetrator groups.

In WH 2012, surprisingly, the depressive symptoms of those being bullied by clients were not statistically different from the other groups. Nevertheless, it still seems that being bullied by a leader is associated with worse psychological health in terms of depressive symptoms than being bullied by clients. In the same sample we found that those who were exposed to bullying by leaders reported significantly more severe depressive symptoms compared to those who were bullied by colleagues. These findings also support the argument concerning the organisational power between the perpetrator and the bullied respondents.

Finally, the current study was carried out among those respondents who reported workplace bullying: 9.7 % in DWECS 2010 and 11.9 % in WH 2012. This prevalence is somewhat higher than the prevalence in other Scandinavian countries, yet it is in line with findings showing an overall 11 % in studies using the self-labelling method with a definition [5]. Furthermore, according to the newest report available [39], the prevalence of workplace bullying in the general working population in Denmark was 11.6 % in 2014, thus our findings are in accordance with the prevalence of workplace bullying observed in other Danish studies.

### Strength and limitations

The major strength of the present study is that it was based on two large, nationwide and representative samples. Furthermore, by analysing and comparing data from two cross-sectional surveys of the general working population, we challenged our own findings instead of accepting the results obtained from one survey, or merging them without thorough preliminary analysis. The comparability of the two data sets are supported in that the prevalence of workplace bullying was similar in the two surveys even though they used slightly different definitions of workplace bullying as well as response alternatives. A final strength is that we used a validated and widely used depression scale.

The study also has limitations. Due to the differences between the two surveys, we excluded respondents being exposed to bullying by multiple perpetrators in WH 2012 from the main analysis, although this analytical choice resulted in lower statistical power.

In addition, the findings of the present study may be even more pronounced in samples from other - non-Scandinavian - countries where the differences in workplace hierarchy are associated with bigger perceived power differences. Our samples represent the Danish general working population and therefore, the findings do not necessarily hold in countries where the ruling organisational culture is essentially different than in Scandinavia, which limits the generalizability of the results.

Furthermore, there could be several explanations for the observed associations between the perpetrator and the MDI score of the targets. First, due to the cross-sectional nature of the study, we cannot establish causal relations between being bullied by various perpetrators and the level of depressive symptoms of the targets. Although being exposed to bullying could explain the targets’ more severe depressive symptoms, it is also well-established in longitudinal studies that mental health problems at baseline are associated with an increased risk of subsequent exposure to bullying [22, 24, 40, 41]. In the present study, when comparing the depressive symptoms of the bullied and non-bullied respondents, we found in both cohorts that bullied respondents had higher scores on the depression scale than non-bullied respondents irrespective of the perpetrator. Furthermore, when comparing respondents who were bullied by only one perpetrator with those who were bullied by multiple perpetrators in WH 2012, the mean MDI scores of those being bullied by multiple perpetrators were higher than of those being bullied by only one perpetrator. These results indicate that non-bullied respondents have better mental health than bullied respondents, and that individuals with a poorer psychological health may be exposed to bullying by more people at their workplace. Nevertheless, based on the nature of cross-sectional study there are no grounds to decide whether the worse psychological health is the result or the cause of (the multiple) bullying experience.

Second, it is also plausible that there is bias in the reporting of who the perpetrators is due to other factors, for example reporting the leader as perpetrator due to a general dissatisfaction with the workplace or work climate.

Third, no information on exposures outside working life such as marital status or major life events, which may have confounded with the reported associations, was available in the cohorts. These factors are well-known risk factors for the development of depression, and if these factors are differentially distributed among the perpetrator groups, they may have biased the differences in depressive symptoms between the groups. However, the direction of the bias (under- or overestimation) is unknown.

Overall, alternative explanations for the differences between the groups could be reverse causality, differential misclassification, and unadjusted confounding.

Furthermore, due to the lack of response categories regarding the frequency of bullying in DWECS 2010, the data did not allow us to assess the health effects of workplace bullying based on the frequency of the exposure. Similarly, there was a difference in the way participants were asked about the perpetrators in the two surveys. In DWECS 2010, respondents could only choose one perpetrator, and therefore in case of experiencing bullying by multiple perpetrators, they had to indicate the perpetrator based on a hierarchy, of which we had no information. Consequently, we cannot be sure that in DWECS 2010 there was only one perceived perpetrator of the targets, which made it impossible to evaluate the depressive symptoms of those who perceived themselves as being exposed to multiple perpetrators. Overall, we cannot rule out that this dissimilarity may have influenced our results in a way that could not be further explored within the scope of the present study.

Finally, not everyone was at risk of being bullied by all perpetrators, although we aimed at including respondents being at risk to be bullied by anyone listed in the two surveys. However, in certain professions it may not be the case to work together with all the potential perpetrators and thus being at risk to be bullied by them. Nevertheless, our data did not let us to eliminate this issue beyond applying our inclusion criteria.

## Conclusion

The results of the present study indicate that the level of depressive symptoms of bullied employees differ according to who the perpetrator is, with a tendency of more severe depressive symptoms among employees who are subjected to bullying by their leaders, and the least severe symptoms among those who are bullied by clients. This paper highlights the importance of considering workplace bullying as a serious problem for individuals experiencing it. As shown, workplace bullying may be perceived the worst when the perpetrator is on top of the formal organisational hierarchy. Thus, leaders must be aware of the consequences of their actions and need to make an effort to avoid becoming perpetrators themselves. Furthermore, due to their organisational power and obligations, leaders should play a crucial role in prevention by developing anti-bullying policies as well as they need to actively intervene in situations where workplace bullying is already unfolded among individuals in order to de-escalate it.

Future prospective studies should aim at testing that the cross-sectional associations found in our study are actually causal. Finally, even though the self-reporting technique could be an obstacle, our results underline that there is a great need for future studies inquiring not only about the targets but also about the perpetrators (e.g. gender and place in the organisational hierarchy) in order to increase the effectiveness of the bullying prevention at workplaces.

## Abbreviations

DWECS 2010: Working Environment Cohort Study
MDI: Major Depression Inventory
UK: United Kingdom
US: United States of America
WH 2012: Work and Health Study
